# Antibiotic treatment and flares of rheumatoid arthritis: a self-controlled case series study analysis using CPRD GOLD

**DOI:** 10.1038/s41598-019-45435-1

**Published:** 2019-06-20

**Authors:** Navraj S. Nagra, Danielle E. Robinson, Ian Douglas, Antonella Delmestri, Stephanie G. Dakin, Sarah J. B. Snelling, Andrew J. Carr, Daniel Prieto-Alhambra

**Affiliations:** 1 Nuffield Department of Orthopaedics, Rheumatology and Musculoskeletal Sciences (NDORMS), University of Oxford, Botnar Research Centre, Old Road, Oxford, UK; 2 Department of Non-Communicable Disease Epidemiology, London School of Hygiene and Tropical Medicine, London, UK

**Keywords:** Epidemiology, Rheumatoid arthritis, Risk factors

## Abstract

There is emerging evidence of the impact of infections on rheumatoid arthritis pathogenesis and flares. We aimed to study the association between antibiotic use (and timing of use), and the occurrence of flares in patients with RA. We nested a self-controlled case series (SCCS) of patients who have RA flares within a newly diagnosed RA cohort (n = 31,992) from the UK Clinical Practice Research Datalink (CPRD) GOLD dataset. We determined associations between exposure to antibiotics (beta-lactam, imidazole, macrolide, nitrofurantoin, quinolone, sulphonamide and trimethoprim, and tetracycline) and the occurrence of RA flares. Conditional fixed-effects Poisson regression models were used to determine incidence rate ratios (IRR), offset by the natural logarithm of risk periods. A total of 1,192 (3.7%) of RA subjects had one or more flare/s during the study period, and were therefore included. Use of sulphonamide and trimethoprim was associated with an increased risk of RA flare at 29–90 days (IRR 1.71, CI 1.12–2.59, p = 0.012); 91–183 days (IRR 1.57, CI 1.06–2.33, p = 0.025); and 184–365 days (IRR 1.44, CI 1.03–2.02, p = 0.033) after commencement of antibiotic treatment. No other antibiotic group/s appear associated with RA flare/s risk. Usage of sulphonamide and trimethoprim antibiotics, is associated with a 70% increased risk of RA flare at 1–3 months, which decreases but remains significant up to 12 months after treatment. We hypothesise that the delayed onset of RA flares after specific antibiotics is mediated through the gut or urinary microbiomes. Further epidemiological and mechanistic research is needed to determine the role of infections in RA.

## Introduction

Rheumatoid arthritis (RA) has a poorly understood aetiology. Genetics, smoking and hormones have all been implicated, however the emerging idea of a microbial trigger (infections of a specific body part by a specific pathogen) has been investigated in greater depth recently^1–3^. The role of epidemiological studies has, historically, been important in generating hypotheses for disease pathogenesis. Previous epidemiological studies have shown positive associations between bacteria such as *P. gingivalis* in periodontitis and incident RA. These studies have instigated a new area of microbiome research in RA pathogenesis, with conflicting findings between candidate microbial triggers and exploratory mechanistic studies^4,5^.

Recent epidemiological studies have shown associations between the occurrence of acute bacterial infections and incident RA, although results are conflicting^6^. Some of these studies have proposed the microbiome as a potential mediator for the effect of infection in RA pathogenesis. A number of antibiotics were originally used as treatments for RA, and the sulphonamide derivative, sulfasalazine, is still used as a DMARD today^2^. Antibiotics have also been shown to significantly perturb the gut and urinary microbiome, with trials showing substantial microbial shifts in the gut lasting up to a year after treatment periods of at least one week^7,8^. Despite knowledge that the microbiome is potentially disrupted in RA and that antibiotics perturb the microbiome, the impact of antibiotic treatment on RA pathogenesis has not been adequately investigated^6,9^. New research has also highlighted the importance of investigating patients during flares of RA symptoms, when triggers of disease may be more evident^10^.

In this hypothesis-generating study, using a self-controlled case series (SCCS) analysis, we determined the association between antibiotic therapy and flares of RA in an established RA population. Our null hypothesis stated that no association would be observed.

## Methods

### Data source

The study was undertaken using data from the UK Clinical Practice Research Datalink (CPRD) GOLD dataset, which contains anonymised electronic medical data of over 15.5 million primary care patients over 740 UK practices and is representative of the national population with regards to age, gender and socioeconomic status^11,12^. Within the UK, the majority of patients will be reviewed and treated by their primary care physician, with only a small proportion of interactions necessitating further review by specialists^13,14^. All interactions are coded in practices which contribute to CPRD. These practices must attain a certain level of quality of coding before sharing information with CPRD. The quality of data is regulated, and the database is managed by the Medicine and Health Products Regulatory Authority (MHRA)^11^.

Data of interest contained in the CPRD includes ‘Read codes’ which cover medical diagnoses (made in primary care or secondary care), patient co-morbidities and clinical measurements such as smoking status, alcohol drinking, or BMI according to a hierarchical clinical classification system; drug prescriptions according to the British National Formulary code; in addition to general demographic data and laboratory test results^15,16^.

### Participants

The target population was identified by clinical or referral events of RA using Read codes occurring from 1987 to February 2015. At start date (first-ever diagnosis of RA), patients had to be registered at the practice for at least one year and also the practice had to be declared up-to-standard for clinical research for at least one year. The target population only included patients who had the first RA diagnosis when they were at least 18 years old. Using these inclusion criteria, 31,992 eligible patients were identified for this study. Previous studies have shown that RA diagnosis has high validity in CPRD GOLD, when assessed against American College of Rheumatology diagnostic criteria^13,14^.

From within the dataset, the diagnosis of flares of RA was determined based on the single Read code, ‘Flare of rheumatoid arthritis’. Of the total 31,992 patients, 1,192 patients were noted to have flares of disease, and were therefore included for all subsequent analyses.

### Outcome and exposures

The outcome of interest was flares of RA, as determined by primary care diagnosis and coding in CPRD Read Codes. It was noted that patients might attend their primary care physician multiple times within the same episode of RA flare, with failure of resolution of symptoms. Therefore, in order to differentiate ongoing symptoms in the same episode, from two discrete episodes, a time period of a minimum of two weeks was set, such that RA flare codes separated by two weeks were deemed separate events, but less than two weeks were considered the same flare episode. The RA flare codes were subdivided further, using a flag for concomitant acute prescriptions of oral, intra-articular or intramuscular glucocorticoid steroids e.g. prednisolone, to determine more ‘severe’ rheumatoid flares treated with steroids. Only prescriptions with a minimum dose of 5 mg were included to prevent incidental inclusion of glucocorticoid treatment for other chronic diseases.

The exposure studied was prescription of antibiotics after RA diagnosis. Antibiotic prescriptions were identified using pre-specified lists of CPRD product codes. Antibiotics were categorised as per the World Health Organisation’s (WHO) Collaborating Centre for Drug Statistics Methodology (codes J01A-G and M), in to: aminoglycosides, amphenicols, beta-lactams (and beta-lactams ‘other’), daptomycin, fosfomycin, glycopeptides, imidazoles, linezolid, macrolides, methanamine, nitrofurantoin, quinolones, spectinomycin, steroidal antibiotics, sulphonamides and trimethoprim, and tetracyclines^17^. Antibiotic preparation was limited to oral and intravenous formulations. The associated date of the prescription and the defined daily dose (DDD) of the prescription was also noted to determine the duration of treatment.

All disease and drug codes were determined using the ‘Code Browser’ v3.0.0, (CPRD Research Applications, UK), and further refined after review by an experienced senior data manager (AD) and an academic general practitioner with extensive expertise in the use of CPRD data (DPA).

### Statistical methods

Comparisons of the demographic variables between the subset of patients with flares coded were made with the non-flare coded patients, including age, gender, smoking status and Charlson Comorbidity Scores at one-year before RA diagnosis (grouped 0, 1, 2, 3–5 and 6+).

**Figure 1:**
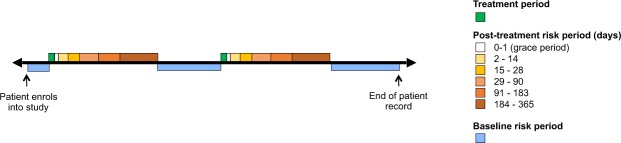
Example schematic of SCCS study type for a patient with two antibiotic treatment periods. Treatment periods (highlighted in green) are the exposures, and varying risk windows are observation periods in to which the outcome of interest (RA flare) may or may not occur. Baseline risk periods account for increasing patient age, and only RA-diagnosed patients were enrolled into the study.

An SCCS was used to determine the association of antibiotic prescription (by category) and subsequent risk of RA flare (Fig. 1). SCCS methodology compares a patient’s probability of having an outcome of interest during a risk period, with a control period^18,19^. The SCCS method looks at individual patients, and the method compares the risk of the outcome of interest occurring at different time-windows within the *same patient’s* observation window. All confounders that remain static over the observation period are intrinsically controlled for whilst those changing over time e.g. age can be controlled for^18^. The method assumes that outcomes do not influence future exposures (in this instance that a flare of RA does not permanently change the likelihood of a future dose of antibiotics), and that events are independent of each other^18–20^.

Risk windows were created in two different patterns. The first series of time windows accounted for changes in the microbiome that has previously been observed in patients treated with different antibiotic groups, with the most dramatic changes occurring in the first two weeks, but with lasting changes occurring at between 6 and 12 months^7,8^. Due to the high sensitivity required in this analysis, a one-day time grace period was given in the second series to account for time taken for patients to obtain antibiotics from their pharmacy. A second series of time-windows was of standardised three-month blocks from the exposure date, leading up to one-year post-exposure in drugs of interest.

A conditional fixed-effects Poisson regression model was used to determine incidence rate ratios (IRR), offset by the natural logarithm of risk periods^18^. Power calculations were performed using specific sample size calculations for SCCS analysis from Musonda *et al*.^21,22^.

Stratified secondary analyses were performed for smoking status, severity of RA flare and length of antibiotic treatment.

The effect of longevity of antibiotic treatment was assessed by performing a sensitivity analyses after dropping observations where treatment was greater than 14 days. The validation of RA flare diagnostic codes was confirmed using a sensitivity analysis in antibiotics of interest, with steroid-linked RA flare episodes compared with non-steroid-linked RA flare episodes.

All analyses were conducted using STATA/IC v15.1 (StataCorp LLC, TX, USA). There was no involvement of patients in the planning or conduct of the study.

### Ethical review

Permission to run the study was given by the Independent Scientific Advisory Committee (ISAC) for MHRA Database Research.

## Results

### Description of RA population

Of the 31,992 adults with a diagnosis of RA within the overall cohort, 1,192 (3.7%) had at least one RA flare episode coded within the study period. A total of 1,621 separate flare episodes were coded. Patient characteristics in the RA flare and the remaining RA cohort are detailed in Table 1. The mean age of the RA cohort at diagnosis was 60.6 (SD 15.0) years and slightly younger for the flare cohort (57.0 (SD 14.3) years). The proportion of women was slightly lower in the overall cohort (68.8%) compared to the RA flare patients (72.6%). BMI was similar between groups, with mean 27.4 (SD 5.7) kg/m^2^ and 27.4 (SD 5.9) overall and in the RA flare population respectively. Smoking status revealed significantly higher ‘Current’ and ‘Ex-smoking’ status in the flare cohort compared to the non-flare cohort. Finally, co-morbidity (as measured using the Charlson Co-morbidities Index) was similar in the flare and non-flare cohorts.

**Table 1:** Demographic data of patients included. Values are taken at time of entry in to study.

	RA cohort (n = 30,800)	RA Flare cohort (n = 1,192)	Flare cohort with concurrent steroid prescriptions (n = 538)
Age at entry (age at RA diagnosis), years, mean ± SD	60.6 (15.0)	57.0 (14.3)	57.7 (14.4)
Women, *n* (%)	21,190 (68.8%)	859 (72.6%)	384 (71.4%)
Body mass index, kg/m^2^, mean ± SD	27.4 (5.7)	27.4 (5.9)	27.8 (6.0)
Range	11.6–59.8	14.1–60	14.5–51.3
Body mass index, kg/m^2^, mean ± SD (at 1 year post entry)	29.0 (6.1)	27.7 (6.0)	28.5 (6.4)
Range (at 1 year post entry)	10.9–59.7	12.4–49.2	14.6–48.3
<20 kg/m^2^, n, (%)	1094 (5.3)	46 (5.9)	22 (5.9)
20–25 kg/m^2^, *n* (%)	6615 (31.9)	233 (29.8)	110 (29.5)
25–30 kg/m^2^, *n* (%)	7310 (35.3)	283 (36.2)	126 (33.8)
>30 kg/m^2^, *n* (%)	5701 (27.5)	220 (28.1)	115 (30.8)
**Smoking status, n (%)**			
Current	5737 (23.0)	288 (30.5)	124 (27.9)
Ex-smoker	7673 (30.8)	264 (28.0)	141 (31.8)
Never smoked	11530 (46.2)	392 (41.5)	179 (40.3)
**Comorbid conditions - Charlson Co-morbidity Score**, ***n*** **(%)**			
0	27,737 (90.0)	1,078 (90.4)	481 (89.4)
1	1930 (6.3)	75 (6.3)	37 (6.9)
2	850 (2.8)	32 (2.7)	15 (2.8)
3, 4 and 5	239 (0.7)	6 (0.5)	<5 (0.7)
6+	39 (0.1)	<5 (0.1)	<5 (0.2)

The majority (79.4%) of patients coded as having RA flares had only one flare during their time in the database, 12.8% had two flares, 4.4% had three, 1.6% had four, and 1.8% had at least five flares (maximum 13). Regional distribution of patient frequency with a flare code is represented in Appendix Fig. 1. For secondary analyses, a subset of potentially more severe flares was identified including 762 (47.0%) coded episodes with an associated steroid prescription in 538 patients. This is subdivided per flare episode in Appendix Table 1. The group of RA flare patients who had a concurrent steroid prescription were also examined and their demographic data is also reported in Table 1.

### Antibiotic usage in the study population

A total of 12,743 antibiotic prescriptions were included within the SCCS analysis, across 1,192 patients. Of all antibiotic groups, only beta-lactam, imidazole, macrolide, nitrofurantoin, quinolone, sulphonamide and trimethoprim, and tetracycline antibiotics had enough prescription episodes for an appropriately powered SCCS analysis. Antibiotic usage, including proportion of patients who had at least one antibiotic prescription, and length of prescriptions as expressed by DDD’s, are described in Table 2. Within the flare cohort, 75.8% of patients had three or fewer antibiotic categories and 0.8% had all seven antibiotic categories over their time in the study (10.7% had no antibiotics, 23.9% had one, 22.8% had two, 18.4% had three, 13.3% had four, 7,6% had five and 2.5% had six).

**Table 2:** A summary of antibiotic usage between the flare and non-flare populations.

	Antibiotic group	Non-flare patients	Flare patients
Antibiotic usage (at least one prescription [%])	Beta-lactam	61.29%	76.01%
	Imidazole	8.56%	11.99%
	Macrolide	33.15%	49.07%
	Nitrofurantoin	12.31%	19.20%
	Quinolone	14.02%	21.48%
	Sulphonamide and Trimethoprim	24.75%	32.21%
	Tetracycline	19.52%	28.27%
Antibiotic usage (total DDD’s [+/− SD])	Beta-lactam	46.29 (+/− 137.41)	62.07 (+/− 134.11)
	Imidazole	12.23 (+/− 16.04)	11.23 (+/− 12.72)
	Macrolide	31.17 (+/− 91.43)	35.47 (+/− 102.81)
	Nitrofurantoin	42.93 (+/− 185.45)	60.71 (+/− 243.65)
	Quinolone	24.49 (+/− 80.50)	27.71 (+/− 83.52)
	Sulphonamide and Trimethoprim	29.91 (+/− 156.68)	28.33 (+/− 97.17)
	Tetracycline	58.26 (+/− 242.83)	56.50 (+/− 177.54)

### Self-controlled case series (SCCS)

**Figure 2:**
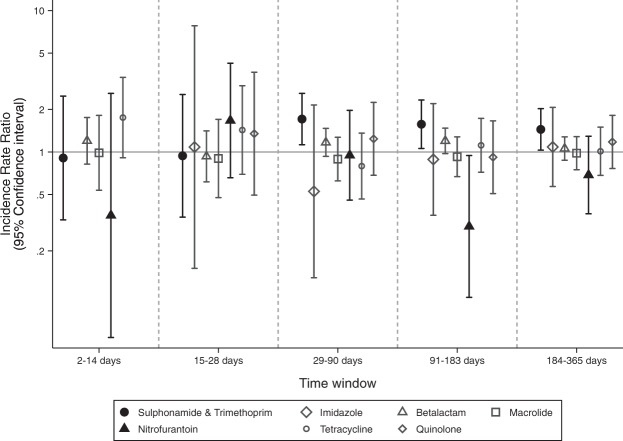
A graph to show the IRR of studied antibiotics (with 95% confidence intervals) for rheumatoid arthritis flare after commencing treatment. For antibiotics with statistically significant results (*p < 0.05), the results are highlighted in black.

Figure 2 shows the results of the SCCS analysis for eligible antibiotics, (specific IRRs are described in Table 3). Sulphonamide and trimethoprim treatment was associated with an increased risk of a flare in risk windows between 29–90 days (1–3 months) by 70.9% (IRR 1.71, CI 1.12–2.59, p = 0.012); between 91–183 days (3–6 months) by 57.0% (IRR 1.57, CI 1.06–2.33, p = 0.025); and between 184–365 days (6–12 months) by 44.4% (IRR 1.44, CI 1.03–2.02, p = 0.033) above baseline risk. No other antibiotic use had an association with excess risk of flare/s.

**Table 3:** Incidence Rate Ratios with 95% Confidence Intervals for flares of rheumatoid arthritis after exposure of antibiotics. Antibiotic groups of interest after analysis are highlighted in italics.

	0–1 days (grace period)			2–14 days			15–28 days			29–90 days			91–183 days			184–365 days		
	IRR	95% Lower CI	95% Higher CI	IRR	95% Lower CI	95% Higher CI	IRR	95% Lower CI	95% Higher CI	IRR	95% Lower CI	95% Higher CI	IRR	95% Lower CI	95% Higher CI	IRR	95% Lower CI	95% Higher CI
*Sulphonamide and Trimethoprim*	0.00	0.00	.	0.91	0.33	2.48	0.94	0.35	2.55	1.71*	**1.13**	**2.60**	1.57*	**1.06**	**2.33**	1.44*	**1.03**	**2.02**
Imidazole	0.00	0.00	.	0.00	0.00	.	1.08	0.15	7.81	0.53	0.13	2.15	0.89	0.36	2.20	1.09	0.57	2.07
Beta-lactam	1.60	0.59	4.32	1.20	0.82	1.75	0.93	0.62	1.41	1.17	0.93	1.47	1.20	0.97	1.47	1.06	0.87	1.28
Macrolide	1.12	0.16	7.97	0.99	0.54	1.81	0.90	0.48	1.70	0.89	0.62	1.27	0.93	0.67	1.28	0.98	0.75	1.28
*Nitrofurantoin*	3.84	0.52	28.16	0.36	0.05	2.60	1.67	0.66	4.24	0.95	0.46	1.97	0.30*	**0.09**	**0.94**	0.69	0.37	1.29
Tetracycline	0.00	0.00	.	1.75	0.91	3.37	1.43	0.70	2.94	0.79	0.46	1.36	1.12	0.72	1.73	1.01	0.68	1.50
Quinolone	0.00	0.00	.	0.00	0.00	.	1.35	0.50	3.66	1.24	0.68	2.24	0.92	0.51	1.66	1.18	0.76	1.81

Time periods when risk of flare was increased or decreased are highlighted in bold (*p < 0.05).

It is noteworthy that, nitrofurantoin antibiotic treatment was associated with a reduced risk of flare, with flare probability between 91–183 days (3–6 months) reduced by 70.2% (IRR 0.30, CI 0.09–0.94, p = 0.04). This association was however not observed for any other time window/s.

Using the secondary three-month time windows from the exposure date, with sulphonamide and trimethoprim antibiotics, there was a significant increased risk of a flare in risk windows between 273–365 days (9–12 months) by 70.3% (IRR 1.70, CI 1.14–2.55, p = 0.01). No other antibiotic use had an association with excess risk of flare/s.

### Sensitivity analyses

Sensitivity analyses with smoking status showed no interaction of smoking on effect size of sulphonamide and trimethoprim antibiotic usage on flare incidence (Appendix Table 2).

A secondary analysis to examine ‘severe flare’ episodes (flare attendances when a steroid was prescribed concurrently within primary care), was performed by deleting RA flare encounters where a steroid was not prescribed. The analysis in antibiotics of interest was underpowered, and although trends were similar to the whole flare cohort analysis, there was no significant interaction between any of the antibiotic groups and flares (Appendix Table 2).

A final sensitivity analysis examined dose related effects. All sulphonamide and trimethoprim prescriptions were divided in to quartiles based on DDD’s and separate SCCS analyses re-run. This analysis was underpowered within each quartile, however trends (increased risk of flare between 1 and 12 months) were similar to that of the whole flare cohort analysis. Of note however, within the third quartile (mean DDD’s 7 SD 0.00) an increased risk of diseases flares reached significance between 185–365 days (6–12 months) by 95.0% (IRR 1.95, CI 1.19–3.16, p = 0.007) (Appendix Table 2).

## Discussion

In this novel hypothesis-generating pharmaco-epidemiology study of antibiotic treatment and risk of RA flare in adults using the CPRD GOLD dataset (UK), the majority of patients had at least had one treatment episode with antibiotics during their time within the study. Rheumatoid arthritis flare coding was validated using concurrent steroid treatment and good association found between these two variables. This study found that the risk of RA flare was significantly increased in the 1–12 months after commencing treatment on sulphonamide and trimethoprim antibiotics.

There was also a negative association noted between nitrofurantoin antibiotics and risk of flare with a protective association between 3–6 months after treatment. These findings are of particular relevance given the growing evidence showing associations between gut and urinary tract microbiomes and pathogenesis and severity of RA.

### Strengths and limitations of this study

Like all retrospective pharmaco-epidemiological studies, there are a combination of strengths and weakness in the study methods and, therefore, the conclusions that can be drawn.

The strengths of this study are important. With 70% of patients being female, a higher proportion of obesity (classified by BMI > 30 kg/m^2^) within our cohort compared to reference values within the CPRD GOLD, the population statistics of the study populace were consistent with previous studies and the wider RA patient population^23–25^. Specifically, within the RA flare cohort, the increased prevalence of current smokers is consistent with other retrospective population studies which show higher flare rates in current smokers^26^. This population is also UK-wide and therefore not affected by local/environmental factors, which may protect against or increase risk of RA flares. This is also controlled for by the SCCS study design.

Secondly, the UK population is strictly controlled with regards to access to antibiotics. Patients are unable to purchase antibiotics ‘over the counter’ (OTC) unlike in other countries, and therefore the accuracy of prescription data within the CPRD GOLD is high. Thirdly, with regards to data accuracy, by evaluating the ‘Read Codes’ for RA flares and determining associated acute high dose steroid prescription, we validated the outcome of interest in the patient group.

Fourthly, the SCCS analysis has significant advantages when compared to other epidemiological study designs. Because all included patients have the outcome of interest, studies can be powered more efficiently and confounding factors are intrinsically controlled for. The analysis also allows specific time windows to be set which fit the hypothesis regarding time taken for citrullination of peptides and autoimmunity to develop, less than 18 months^27^. One-year time windows were used as current best-evidence shows in patients who have antibiotics which most radically change the microbiome, e.g. ciprofloxacin, the faecal flora are largely recovered by the 1-year time point^8^. We would therefore expect to capture prolonged autoimmune effects mediated by the microbiome.

The limitations of this study, and the conclusions that can be drawn from it, are important. First, CPRD GOLD data will not capture all patient flare episodes, thus limiting power of the analysis. Within the UK National Health Service, there are several pathways by which patients can access care such as, direct access to specialist nurses or dedicated ‘flare clinics’ in secondary care. Our study did not include such episodes and it is unclear as to whether this would bias our patient population, since provision of RA flare clinics will vary by region. Secondly, antibiotic usage via secondary care, dental care and other community sources (approximating 26% of total antibiotic prescriptions) was not accounted for in this study^28^. This could mean we underestimated the effect of antibiotic groups on flares.

Thirdly, this study was not powered for all sub-analyses and for other potentially interesting questions such as antibiotic interactions and compounding effects of different antibiotics. One other such sub-analysis centres on evolution of treatments over time, especially since the first patients were enrolled in to this study, specifically with the advent of biological DMARDs. With the approximate introduction of biologics in the year 2000^29^, we note approximately 10% of the flares recorded in our dataset would be from a ‘pre-biologics’ era. The potential effect on the SCCS analysis is difficult to interpret, however it can be assumed the baseline level of risk of flare within the SCCS model will account for changes in patient medication.

Finally, to examine the effect of antibiotics on RA flares in an unbiased manner, the indication for the antibiotic prescription was not investigated, and therefore we cannot exclude the role of the infection in the association with RA.

### Findings in the context of the literature

Previous studies have looked at the role of infections in triggering and propagating RA, specifically, urinary tract infections (UTIs) have been implicated with first diagnosis of RA. However, the evidence for this relationship is inconsistent.

The pathophysiology of RA flares specifically, and whether this is distinct from index RA, is not fully understood, however an understanding of the processes that cause RA can give greater context to findings. Briefly, RA can be separated in to two subgroups depending on the presence of pathological anti-citrullinated protein antibodies (ACPAs). These are antibodies against proteins (e.g. fibrin, type II collagen and fibronectin, amongst others) which have undergone citrullination, a physiological post-translation modification of arginine groups. The majority of patients with RA have ACPA-positive RA, and the presence of ACPAs is 97% specific to RA, underlining its importance as a hallmark of RA disease^30^. This is further supported by genetic risk factors for RA largely being related to genes involved in production of ACPAs. Consideration should also be given to other studies have shown that antibodies against bacterial species e.g. *Proteus mirabilis* have cross reactivity with self-antigens found in collagen type XI, so-called ‘molecular mimicry’^31^. The role of the immune system in RA is now better understood. T-lymphocytes are activated against self-antigens or citrullinated proteins which subsequently (after co-stimulation via CD28) proliferate and release cytokines to activate B-lymphocytes^31^. B-lymphocytes then terminally differentiate to plasma cells and release autoantibodies. This eventually results in activation of macrophages, neutrophils and native cells within the joint (such as synovial fibroblasts and chondrocytes) to mediate the joint damage observed in RA through a release of inflammatory cytokines, oxygen derived free radicals and proteases and collagenases^30^. Of relevance, murine studies have shown indications that flares of RA could be mediated by T-lymphocytes through an IL-23 dependent manner, however further work and correlation to human disease is required^32^.

In the context of the microbiome, a number of bacteria have been implicated in the process of citrullination (*Porphyromonas gingivalis*, a bacterium associated with periodontitis)^33^, the hypercitrullination of neutrophils as part of the immune system response to such proteins (*Aggregatibacter actinomycetemcomitans*)^34^, in ‘molecular mimicry’ (*Escherichia coli* and *Proteus mirabilis*)^31^ and in the production of antibodies by B-lymphocytes which mediate the autoimmune response (Epstein-Barr virus)^35^.

Focussing on previous hypotheses that UTIs with the specific bacterium, *Proteus mirabilis* can trigger autoimmunity, auto-antibodies have been reported in RA cohorts from 16 different countries^9^. Authors of such studies have previously recommended starting RA patients on anti-*Proteus* antibiotics^9^. Conversely, findings from Sandberg *et al*. which state UTIs (or rather, the antibiotic treatment of them) may be protective against RA^6^. In their study of patients diagnosed with RA and healthy matched controls, those with RA were more likely to have had a gastrointestinal or genitourinary infection in the two years preceding RA diagnosis. The study was limited on the grounds that antibiotic treatment data for infections (apart from respiratory infections) was not collected.

These findings are of interest because antibiotics that had significant associations with RA flares in our study are primarily prescribed for UTIs. Sulphonamide and trimethoprim antibiotics for UTIs, have good action against *Proteus*, and had a harmful association with RA flares in our study^27^. Although, it could be overly simplistic to consider that treatment with a broad group of antibiotics will always reduce RA flares. In reality, flares of RA are likely a multifaceted disease process, with trauma and lifestyle factors implicated^36^. However, in the case of a potential bacterial trigger, we must also consider the fact that should certain bacteria be implicated in flares of RA, growing resistance to antibiotics, especially in the case of UTIs, will complicate treatment. Evidence summarised by Schaffer *et al*. has shown that *Proteus mirabilis* resistance to sulphonamides and trimethoprim is as high as 83% in some regions^37^.

In either instance, associations do not imply causation; it is unclear if the infection itself triggers the cascade of events which cause a flare in disease, before patients become symptomatic and require antibiotic treatment of the bacterial infection i.e. the antibiotic prescription is a surrogate measure for infection. An alternative theory is that sulphonamide and trimethoprim antibiotics are acting through another microbial trigger, as yet unidentified.

Of note, nitrofurantoin antibiotics are also commonly prescribed in UTIs, but they do not have any effect against *Proteus* species^27,38^. This adds weight to a potential alternative microbial trigger, given the protective association of nitrofurantoin that was observed in this study, which the authors accept could be a spurious finding.

In addition to the genitourinary region, the gut microbiome has also been considered as a site which is implicated in RA pathogenesis^27^. Recently, rare lineages of bacteria have been observed in RA patients within the gut, and dietary factors have also been associated with a reduced risk of developing RA^39,40^. These findings suggest the microbiome profile or signature has a part to play in RA pathogenesis/flares of disease, rather than one specific bacteria^30^. One such study used metagenomic shotgun sequencing and a metagenome-wide association (MGWAS) to show that patients with RA have significantly different oral and gut microbiomes compared to healthy controls, but also, that treatment of RA partially restores the microbiome toward healthy controls^5^. Interestingly, findings of bacterial triggers are echoed by patients themselves. One patient questionnaire-based study linked infections and antibiotic use with RA flares and RA remission respectively. In the study of 274 patients with diagnosed RA, 49.6% believed that an infection precipitated a flare of disease^40,41^. Nitrofurantoin has been shown to alter the gut microbiome in the acute phase, with significant phenotypic changes at least 28 days after the end of treatment^42^. At the time of writing the authors could not find substantial evidence of how sulphonamide and trimethoprim antibiotics alter the human gut microbiome and therefore we cannot comment on this potential mediation (although this shift was evident in a murine model^42^). This is important because immune priming at the gut mediated by perturbed gut bacterial populations following antibiotic use could influence risk of RA pathogenesis and disease flares.

In the broader context of rheumatic diseases, other conditions have been thought to be associated with the microbiome and carriage of certain bacteria. Specifically, granulomatosis with polyangiitis (GPA). Granulomatosis with polyangiitis is a chronic, relapsing, autoimmune condition where antibodies against antigens contained in the granules of neutrophils and monocytes are generated, after an initial inflammatory event. It typically results in vascular inflammation. This condition is highly relevant as the microbiome has been found to be significantly different in GPA patients compared to healthy controls, and *Staphylococcus aureus* species have been associated with relapses of GPA in patients^43^. This association was tested in a prospective randomised control trial of GPA patients who were given either trimethoprim-sulfamethoxazole or a placebo drug for 24 months^44,45^. The study found a significant reduction in relapse rate in the treated group compared to controls (18% vs 40% respectively), which suggests that an anti-bacterial action through co-trimoxazole reduces diseases relapse^45^. Further ‘real world’ methods to test such a hypothesis could be to determine the incidence of disease flares in patients with inflammatory rheumatic diseases given cotrimoxazole prophylaxis for *Pneumocystis* species pneumonia (PCP). A number of studies have shown that prophylactic use of the antibiotic reduces the incidence and mortality associated with PCP in patients with inflammatory rheumatic diseases, particularly in those receiving high steroid doses^46–48^. Unfortunately, no data was collected on patients’ rheumatoid disease outcomes in these studies, including the frequency of flares of disease.

This study has generated directions for future work. This study should be repeated in another dataset from a different patient population to determine whether the effects and their respective magnitudes remain. Furthermore, questions of causality might be addressed by repeating the study using primary care diagnoses of community acquired infections e.g. urinary tract infections or lower respiratory tract infections as the exposure within the methodology. Studies should also look to link with hospital data to determine whether a further 18% of antibiotic prescriptions that are not captured in CPRD GOLD data affect analysis results^28^.

Importantly, this study generated appropriate power for analyses through grouping antibiotics by the WHO ATC/DDD index and further questions around specific antibiotics would also be of clinical relevance. This would allow mechanistic studies to be established to determine whether bacterial targets of these antibiotics are important in disease pathogenesis.

In conclusion, we have found a positive association between sulphonamide and trimethoprim antibiotics and flares of RA, highlighting the importance of considering the treatment of the infection, not solely the infection itself in the disease activity of RA.

## Supplementary information

Supplementary Information
