# Supplementary material for: Antibiotic treatment and flares of rheumatoid arthritis: a self-controlled case series study analysis using CPRD GOLD
Source: Sci Rep. 2019 Jun 20;9:8941. doi: 10.1038/s41598-019-45435-1 (PMC6586671; doi:10.1038/s41598-019-45435-1)

Antibiotic treatment and flares of rheumatoid arthritis: a self-controlled case series study analysis using CPRD GOLD.

Navraj S Nagra[1], Danielle E Robinson[1], Ian Douglas[2], Antonella Delmestri[1], Stephanie G Dakin[1], Sarah JB Snelling[1], Andrew J Carr[1] and Daniel Prieto-Alhambra[1]

[1]Nuffield Department of Orthopaedics, Rheumatology and Musculoskeletal Sciences (NDORMS), University of Oxford, Botnar Research Centre, Old Road, Oxford, UK.

[2]Department of Non-Communicable Disease Epidemiology, London School of Hygiene and Tropical Medicine, London, UK

Corresponding author contact: Prof Daniel Prieto-Alhambra

E-mail: daniel.prietoalhambra@ndorms.ox.ac.uk

Appendix Table 1: A summary of steroid treatment by flare episode number, defined as a minimum of 5mg of glucocorticoid prescribed within one-week of the patient having an RA flare coded. In total, 47.0% of flare episodes had a concurrent steroid prescribed.

Flare episode number	Steroid prescription	No steroid prescription	Total patient number	Percentage (%) Steroid Rx/ Flare Episode
1	538	654	1192	45.1%
2	118	127	245	48.2%
3	52	40	92	56.5%
4	25	15	40	62.5%
5	11	10	21	52.4%
6+	18	13	31	58.1%
Totals	**762**	**859**	**1621**	**47.0%**

Appendix Table 2: Incidence Rate Ratios with 95% Confidence Intervals for flares of rheumatoid arthritis after exposure of sulphonamide and trimethoprim antibiotics, with sensitivity analyses performed for smoking status, severe flare episodes and length of antibiotic course by quartiles (defined daily dose/quartile). Time periods when risk of flare was increased or decreased are highlighted in bold (*p<0.05, **p<0.01).

**Sensitivity analyses category**	**0-1 days (grace period)**			**2- 14 days**			**15-28 days**			**29-90 days**			**91- 183 days**			**184-365 days**		
	**IRR**	**95% Lower CI**	**95% Higher CI**	**IRR**	**95% Lower CI**	**95% Higher CI**	**IRR**	**95% Lower CI**	**95% Higher CI**	**IRR**	**95% Lower CI**	**95% Higher CI**	**IRR**	**95% Lower CI**	**95% Higher CI**	**IRR**	**95% Lower CI**	**95% Higher CI**
**Smoking (current)**	0.00	0.00	.	1.47	0.35	6.07	1.38	0.33	5.76	1.10	0.44	2.78	1.28	0.58	2.83	1.72	0.97	3.06
**Smoking (never)**	0.99	0.00	.	0.94	0.08	10.81	0.00	0.00	.	2.42	0.73	8.03	0.75	0.20	2.76	0.60	0.22	1.61
**Flare cohort with concurrent steroid prescriptions**	0.00	0.00	.	0.91	0.22	3.72	0.00	0.00	.	1.24	0.64	2.39	0.41	0.15	1.13	1.37	0.86	2.18
**DDD 1st Q**	0.00	0.00	.	0.71	0.10	5.07	0.67	0.09	4.79	1.39	0.67	2.80	1.12	0.55	2.30	1.41	0.85	2.36
**DDD 2nd Q**	0.00	0.00	.	1.94	0.47	7.94	0.91	0.13	6.58	1.41	0.61	3.24	0.71	0.26	1.94	1.06	0.55	2.04
DDD 3rd Q	0.00	0.00	.	0.00	0.00	.	0.77	0.11	5.59	1.94	0.97	3.85	1.41	0.71	2.82	**1.95****	**1.19**	**3.16**
**DDD 4th Q**	0.00	0.00	.	1.95	0.24	15.64	1.91	0.23	15.41	0.00	0.00	.	1.57	0.34	7.15	2.15	0.70	6.56

Appendix Figure 1:

Representation of geographical spread of patients within SCCS analysis cohort. Percentages by region reflect proportion of patients with RA flares who have been treated with steroids during at least one of these episodes.

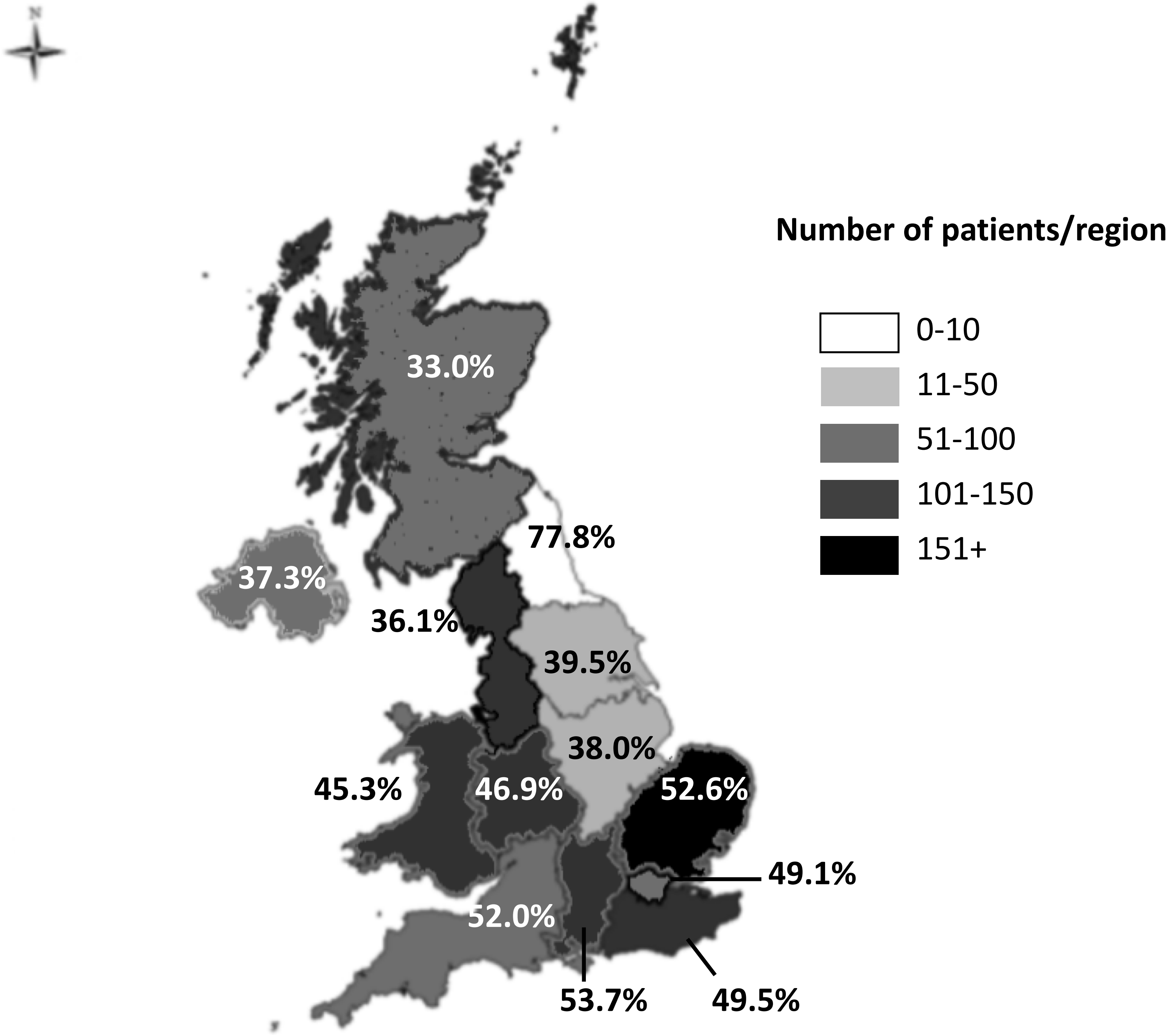

Supplement: Supplementary file 1 — Supplementary Information [file 41598_2019_45435_MOESM1_ESM.pdf]
